# Azvudine Suppresses Epithelial–Mesenchymal Transition in Hepatocellular Carcinoma by Targeting the Notch–HEY Signalling Pathway

**DOI:** 10.3390/ijms26115127

**Published:** 2025-05-27

**Authors:** Yao Meng, Peiyi Sun, Yixin Ren, Guoqing Li, Xiujun Liu, Chunjie Xu, Luyao Dong, Hanhan Li, Zhonghui Zheng, Xuefu You, Xinyi Yang

**Affiliations:** 1 Beijing Key Laboratory of Technology and Application for Anti-Infective New Drugs Research and Development/Laboratory of Pharmacology, Institute of Medicinal Biotechnology, Chinese Academy of Medical Sciences & Peking Union Medical College, Beijing 100050, China; mengyao@imb.pumc.edu.cn (Y.M.); sunpeiyi@imb.pumc.edu.cn (P.S.); gli@imb.cams.cn (G.L.); xuchunjie@imb.pumc.edu.cn (C.X.); dongluyao@imb.pumc.edu.cn (L.D.); 13851868815@163.com (H.L.); zhengzhonghui@sina.com (Z.Z.); 2 State Key Laboratory of Bioactive Substances and Function of Natural Medicines, Institute of Medicinal Biotechnology, Chinese Academy of Medical Sciences & Peking Union Medical College, Beijing 100050, China; liuxiujun2000@imb.pumc.edu.cn; 3 School of Pharmacy, Minzu University of China, Beijing 100081, China; 22400163@muc.edu.cn

**Keywords:** Notch signalling pathway, HEY proteins, EMT, FNC, HCC treatment

## Abstract

Azvudine (FNC) is a novel cytidine analogue that is widely used in the treatment of infectious diseases such as AIDS and COVID-19. Previous studies have demonstrated its anticancer activity in various cancer cell lines, including non-Hodgkin’s lymphomas and lung adenocarcinoma cell lines. However, its effects on hepatocellular carcinoma (HCC) and the underlying mechanisms remain unclear. This study aimed to investigate the anti-epithelial–mesenchymal transition (anti-EMT) activity of FNC and evaluate its potential application in HCC treatment. We found that FNC significantly inhibits the migration of the liver cancer cell line Huh7 by downregulating key EMT markers, such as matrix metalloproteinases (MMPs) and N-cadherin, at both the transcriptional and protein expression levels. Notably, we found that FNC inhibits HEY proteins, particularly HEY1, a transcriptional regulator of the Notch signalling pathway that is overexpressed in approximately 50% of HCC patients. To identify the primary target of FNC, microscale thermophoresis (MST) and molecular dynamics (MD) simulations were performed, revealing that FNC directly binds to Jagged1. This study provides valuable insights into the therapeutic potential of FNC in HCC treatment and elucidates its underlying mechanisms.

## 1. Introduction

Hepatocellular carcinoma (HCC) is one of the deadliest cancers globally. Its incidence has increased over the last few decades, making it the third leading cause of cancer-related deaths [1]. The pathogenesis of HCC involves multi-step molecular changes at both the somatic genomic and epigenetic levels. Knowledge of its molecular mechanisms has expanded rapidly, driven by the development of novel high-throughput technologies, such as the next-generation sequencing, over the past decade [1,2,3,4,5]. However, the most prevalent mutational drivers in HCC remain undruggable [2,4]. Furthermore, the application of molecular pathogenesis knowledge to guide precision diagnosis and systemic therapies for patients is still under investigation [2]. As a result, HCC patients continue to have a very poor prognosis. To date, only two first-line drugs, sorafenib and lenvatinib, have been approved by the FDA for HCC treatment [4]. Although advanced therapies are being rapidly developed, there is a pressing need for more effective therapeutic strategies to address this unmet clinical challenge.

Epithelial–mesenchymal transition (EMT) is a reversible cellular process in which epithelial cells lose their characteristics and acquire mesenchymal traits. During EMT, cells activate families of EMT-associated transcription factors, such as Snail/Slug family and matrix metalloproteinases (MMPs), which enable increased cellular migration [6,7]. Cells can transiently or stably acquire various intermediate states between epithelial and mesenchymal phenotypes (partial EMT) or undergo a complete EMT depending on the cell type and context. EMT is an evolutionarily conserved process involved in both normal development and pathological conditions, including tumour cell migration and invasion [6,8]. It is considered the initial step in the metastatic cascade of cancer, which can be triggered by inflammation, carcinogens or specific features of the local microenvironment of the primary tumour [7,9]. Recent studies have shown that loss of the epithelial phenotype through EMT promotes the acquisition of stem-like traits and resistance to anticancer drugs in carcinoma cells [10]. As such, targeting EMT has emerged as an attractive therapeutic approach, offering potential new avenues for cancer treatment.

The EMT process involves the activation of multiple signalling pathways, including the Notch pathway. The Notch signalling pathway is highly conserved in metazoan species. Both ligands (Jagged1, Jagged2, DLL1 and DLL4) and receptors (Notch1-4) are single-pass transmembrane proteins, and activation occurs through interactions between neighbouring cells. Upon ligand binding, Notch receptors are sequentially cleaved by ADAM family metalloproteases and γ-secretase, releasing the Notch intracellular domain (NICD) [11]. The NICD translocates to the nucleus, where it recruits coactivators to regulate the expression of transcriptional regulators such as the hairy and enhancer of split (HES) and Hes-related family bHLH transcription factor with YRPW motif (HEY) families. The HEY family consists of three members (HEY1, HEY2 and HEYL) in humans, which play critical roles in many developmental processes and the EMT pathway [12]. Overexpression of HEY factors has been associated with advanced tumour progression and poor overall survival, and this has been mostly linked to EMT [13]. HEY transcription factors are highly expressed in malignant carcinomas, such as osteosarcoma [14]. Notably, HEY1 has been reported to be upregulated in 42.6% of HCC tumours in a cohort of 58 samples [15] and in 72.4% of cases in an expanded in-house cohort of 87 HCC patients [13]. HEY1 has also been implicated in regulating EMT in various cell types, making it a promising therapeutic target [12,14]. Inhibiting HEY1 has thus been proposed as a novel therapeutic strategy for cancer treatment.

Azvudine (FNC), also known as 2′-deoxy-2′-β-fluoro-4′-azidocytidine, is a novel cytidine analogue that was initially developed for treating hepatitis C virus (HCV) infection [16,17]. It has been clinically applied in the treatment of HIV and SARS-CoV-2 infection due to its antiviral activity [18,19,20,21]. Recent studies have demonstrated that FNC exhibits antitumour activity in various cell lines and xenograft animal models [17,22,23,24]. In various cancer cell lines, such as B-cell non-Hodgkin’s lymphomas and lung adenocarcinoma, FNC has shown significant effects in suppressing cell proliferation and tumour growth [22,23,25]. Additionally, FNC demonstrated inhibitory activity against adhesion, migration and invasion of non-Hodgkin lymphoma and non-small cell lung cancer cell lines [22,24]. Recently, FNC has been found to induce reactive oxygen species (ROS) production in Dalton’s lymphoma cells [26,27].

Nucleoside analogues have been clinically utilized for over 50 years and remain a cornerstone of cancer treatment [28,29,30]. In HCC studies, nucleoside analogues showed significant efficacy in treating patients with chronic hepatitis B virus (HBV) infections [30,31,32,33,34]. In this study, we aimed to investigate the role of FNC in HCC and elucidate its molecular mechanisms in inhibiting the EMT process in liver cancer cells. Our findings provide critical insights into its potential application in HCC treatment, particularly in inhibiting tumour metastasis, to support its development as a promising candidate for HCC treatment, either as a monotherapy or in combination regiments.

## 2. Results

### 2.1. FNC Inhibits the Migration and Invasion of Huh7 Cells

**Figure 1:**
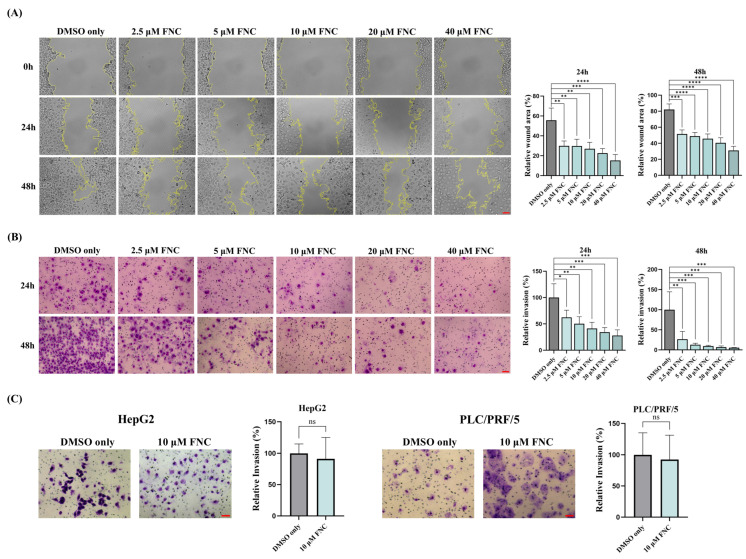
Impacts of FNC on cell invasion of various HCC cell lines. (**A**) Effect of FNC on mobility of Huh7 cells. Representative images of wound-healing assay at 0, 24 and 48 h are shown. Wound surface area measurements are presented as percentages relative to values at 0 h. Values are presented as mean ± SD from three independent experiments. ** *p* ≤ 0.01; *** *p* ≤ 0.001; **** *p* ≤ 0.0001. Scale bar: 20 μm. (**B**) Inhibitory effect of FNC on Huh7 cell invasion. Three independent experiments were performed, and representative images at 24 and 48 h are shown. Results are expressed as mean ± SD relative to control values. * *p* ≤ 0.05; ** *p* ≤ 0.01; *** *p* ≤ 0.001. Scale bar: 20 μm. (**C**) Effect of FNC on invasion capacity of HepG2 and PLC/PRF/5 cells. Three independent experiments were conducted, and representative images captured at 24 h are presented. Values shown are mean ± SD normalized to control. No significant inhibition of invasion was observed with 10 μM FNC treatment in HepG2 and PLC/PRF/5 cells. Comparisons between two groups were performed using a two-tailed unpaired *t*-test. ns, no significant difference. Scale bar: 20 μm.

To determine whether FNC inhibits HCC cell invasion and migration, we performed dose–response wound-healing and transwell invasion assays on Huh7 cells for 24 and 48 h (Figure 1A,B). The scratch wound-healing assay showed that FNC significantly decreased the migration of Huh7 cells in a dose-dependent manner at 24 h (*p* = 0.0042, 0.0040, 0.0018, 0.0006 and <0.0001 for increasing concentrations), with all doses showing significant inhibition at 48 h (Figure 1A). Similarly, the transwell invasion assays demonstrated dose-dependent inhibition at both 24 h (*p* = 0.0391, 0.0070, 0.0020, 0.0008 and 0.0004) and 48 h (*p* = 0.0035, 0.0008, 0.0006, 0.0005 and 0.0004) (Figure 1B). Since 10 μM FNC showed an obvious effect on Huh7 cell invasion, we further assess its impact on additional HCC cell lines (HepG2 and PLC/PRF/5) using transwell assays at this concentration. However, 10 μM FNC did not significantly suppress invasion in either HepG2 or PLC/PRF/5 cells (Figure 1C).

**Figure 2:**
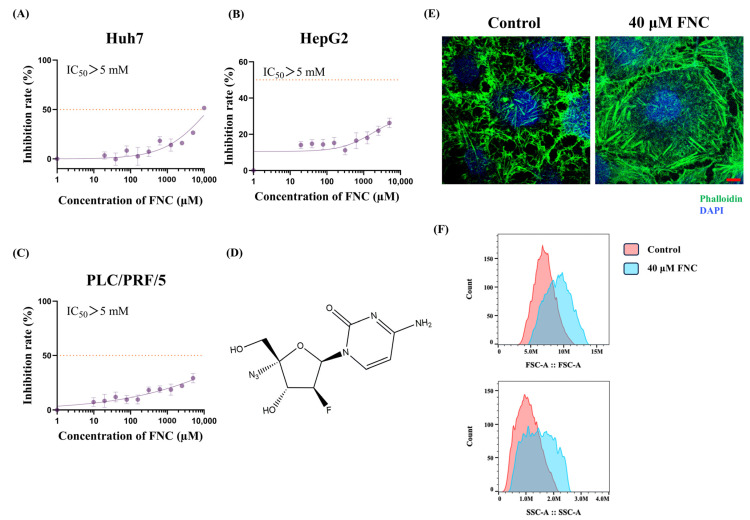
FNC’s cytotoxic and morphological impacts on HCC cells. (**A**–**C**) Cytotoxicity assay results demonstrating effects of FNC on Huh7 (**A**), HepG2 (**B**) and PLC/PRF/5 (**C**) cells. HCC cells were treated with varying concentrations of FNC or DMSO for 48 h, and cell viability was analysed using a CCK-8 colourimetric assay. The orange dotted line indicates the IC_50_ (half-maximal inhibitory concentration). Data are presented as mean ± SEM of three independent experiments. (**D**) Chemical structure of FNC. (**E**) Immunofluorescence microscopy images of Huh7 cells treated with DMSO (control) or 40 μM FNC. Cells were stained with DAPI (blue) to visualize nuclei and phalloidin (green) to stain actin filaments. Images are representative of three independent experiments. Scale bar: 100 µm. (**F**) Flow cytometry analysis of Huh7 cells treated with DMSO (control) or 40 μM FNC for 48 h showing alterations in forward scatter (FSC; cell size) and side scatter (SSC; granularity/complexity) profiles. Data shown are from one representative experiment of three independent biological replicates.

Cell viability in the HCC cell lines was evaluated using the MTT assay after exposure to varying concentrations of FNC for 48 h. The data revealed that FNC exhibited low cytotoxicity in Huh7, HepG2 and PLC/PRF/5 cells, with an IC_50_ > 5 mM (Figure 2A–C). These data suggest that FNC may affect Huh7 cell migration behaviour without exhibiting significant cytotoxicity at the tested concentrations. Fluorescent microscopy was used to assess the morphology of the Huh7 cells, which were stained for nucleus and actin distribution using DAPI and phalloidin, respectively. The data showed that FNC-treated Huh7 cells appeared large, rounded and flat while cells in the control group displayed a smaller and hexagonal shape (Figure 2E). Consistent with these observations, flow cytometry revealed altered forward scatter (FSC) and side scatter (SSC) profiles, further confirming the FNC-induced changes in cell morphology (Figure 2F). Since the molecular mechanisms underlying the anticancer effects of FNC on HCC cell lines, particularly from the perspective of EMT, remain unexplored, we aimed to investigate the potential of FNC as an antimetastatic agent in HCC.

Altogether, these results demonstrate that FNC inhibits Huh7 cell migration and invasion in a concentration-dependent manner while inducing significant morphological changes. Therefore, we hypothesized that FNC might regulate the EMT process in HCC.

### 2.2. FNC Modulates a Subset of EMT Markers in Huh7 Cells

**Figure 3:**
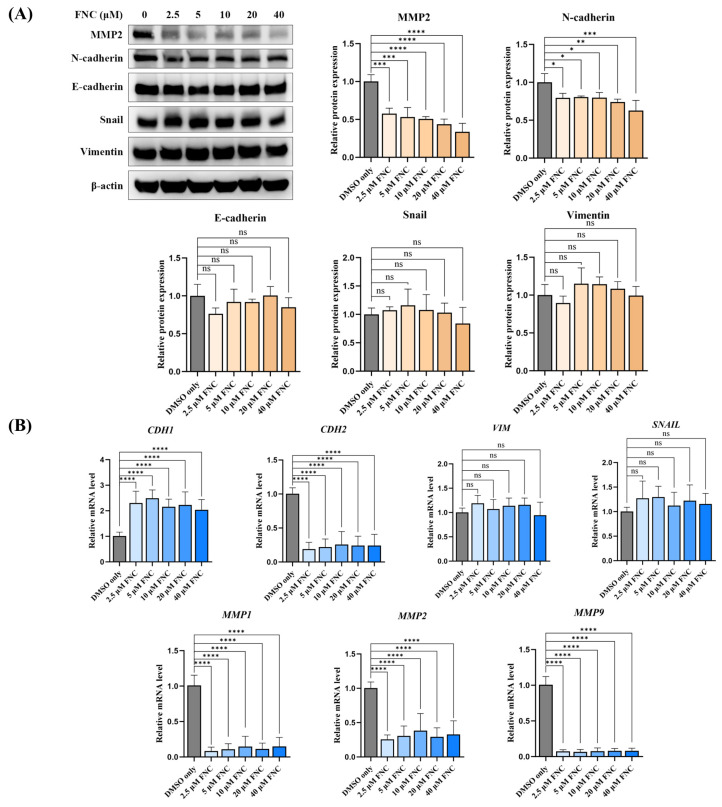
FNC alters a subset of EMT markers in Huh7 cells. (**A**) Immunoblot analysis showing the expression levels of EMT markers, including N-cadherin, E-cadherin, MMP2, Snail and Vimentin, in Huh7 cells after 48 h of FNC treatment. Only DMSO was used to treat the control group. β-actin was used as the loading control. A representative blot from three independent experiments is shown. Protein expression levels were analysed using ImageJ (v2.14.0/1.54f) and are presented as the mean ± SD. ns, no significant difference; * *p* ≤ 0.05; ** *p* ≤ 0.01; *** *p* ≤ 0.001; **** *p* ≤ 0.0001. (**B**) Expression levels of EMT marker genes relative to GAPDH in Huh7 cells, analysed by real-time PCR. Values are presented together with the mean ± SD. ns, no significant difference; **** *p* ≤ 0.0001.

The observed inhibitory effect of FNC on Huh7 cell invasion and migration prompted us to investigate its impact on the transcription and expression of EMT markers. To examine to which EMT markers are regulated by FNC, Huh7 cells were cultured in medium containing FNC at concentrations of 2.5, 5, 10, 20 and 40 μM for 48 h, with DMSO-treated cells serving as the control group. The transcript level analysis revealed that FNC significantly increased the mRNA expression of E-cadherin (CDH1), an epithelial marker and cell adhesion molecule, while decreasing the mRNA expression of N-cadherin (CDH2), a mesenchymal marker associated with the acquisition of an aggressive tumour phenotype (Figure 3B). However, only N-cadherin showed changes in protein levels compared to the control (Figure 3A). MMP2, which is involved in extracellular matrix (ECM) degradation and cell motility regulation, was dramatically reduced at both the transcriptional and translational levels relative to the control group (Figure 3A,B). Notably, the protein-level changes of MMP2 exhibited a dose-dependent trend (Figure 3A). Moreover, MMP1 and 9 showed decreased transcriptional levels after 48 h of FNC treatment (Figure 3B). However, no significant changes were observed in the transcriptional or translational levels of the mesenchymal marker Vimentin (VIM) or the EMT-related transcriptional repressor Snail (SNAIL) (Figure 3A,B). Altogether, these data suggest that FNC partially inhibits the EMT process in Huh7 cells.

### 2.3. RNA-Seq Reveals That FNC May Attenuate Liver Diseases and Function

**Figure 4:**
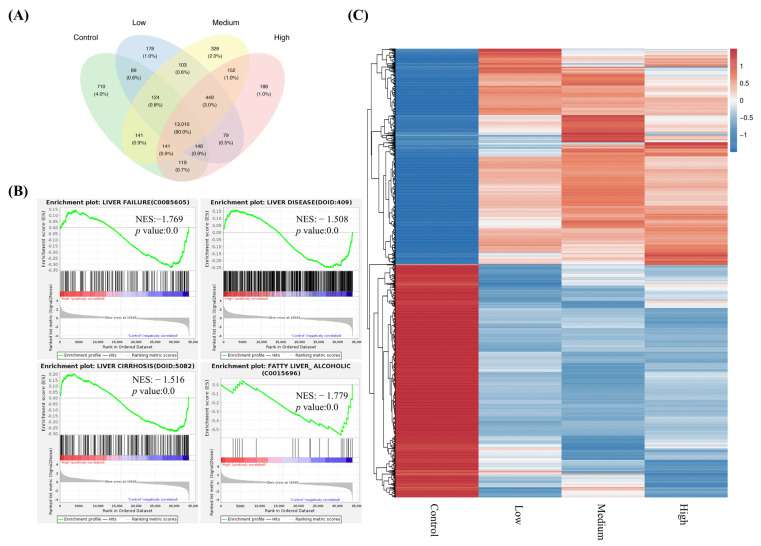
FNC significantly altered the transcriptional profile of Huh7 cells. (**A**) Venn diagram showing the number of genes identified across the different RNA-Seq data comparisons. The green area represents genes from the control group (Huh7 cells treated with DMSO for 48 h). The blue, yellow and pink areas represent genes from Huh7 cells treated with 2.5 μM (low), 20 μM (medium) and 40 μM (high) FNC, respectively. Numbers in the overlapping regions indicate the number of genes shared between multiple groups. (**B**) Gene set enrichment analysis (GSEA) showing significant enrichment of liver disease and liver failure gene sets in the control group compared to Huh7 cells treated with 40 μM FNC. The GSEA enrichment plots illustrate the distribution of the enrichment score (green line) across the genes associated with the respective diseases (vertical black lines) ranked by their anti-disease activity (left to right). Liver failure (*p* value = 0.0; NES = −1.769), liver disease (*p* value = 0.0; NES = −1.508), liver cirrhosis (*p* value = 0.0; NES = −1.516) and fatty liver (alcoholic) (*p* value = 0.0; NES = −1.779) were identified as significantly enriched targets. (**C**) Hierarchical clustering of differentially expressed genes in the control, low-, medium- and high-concentration groups of FNC-treated Huh7 cells. The heatmap uses a colour scale with blue representing the lowest expression and red indicating the highest expression.

To examine the transcriptional effects of FNC on Huh7 cells, RNA-Seq was performed on samples treated with 2.5, 20 and 40 μM FNC, as well as on a control group (Huh7 cells treated with DMSO only). Hierarchical clustering of the RNA-Seq data revealed significant differences in the expression patterns between FNC-treated cells and the control group (Figure 4C). Principal component analysis (PCA) two-dimensional score plots for the control, low-, medium- and high-concentration groups are displayed in Figure S1. The FNC-treated groups were distinctly separated from the control group, suggesting transcriptional disturbances in Huh7 cells following FNC treatment.

The differentially expressed genes and number of overlapping genes among the four groups are shown in Figure 4A. The Venn diagram revealed that 80% of the genes were shared across all four groups. A total of 449 genes, accounting for 3% of the detected genes, were expressed exclusively in all drug-treated groups but not in the control group. The low-, medium- and high-concentration groups uniquely expressed 178 (1.0%), 326 (2.0%) and 188 (1.0%) genes, respectively. Given the anti-EMT effects observed in FNC-treated Huh7 cells in vitro, we conducted gene set enrichment analysis to determine whether FNC affects liver disease-associated gene expression. Notably, liver disease and liver failure gene sets were significantly enriched in the control group compared to the 40 μM FNC-treated group (Figure 4B). Additionally, the liver cirrhosis and alcoholic fatty liver gene sets, which are common features of HCC [1], were significantly downregulated in FNC-treated cells (Figure 4B). These findings suggest that FNC may uniquely inhibit genes associated with liver diseases.

### 2.4. HEY Family Members Are Downregulated in FNC-Treated HCC Cells

**Figure 5:**
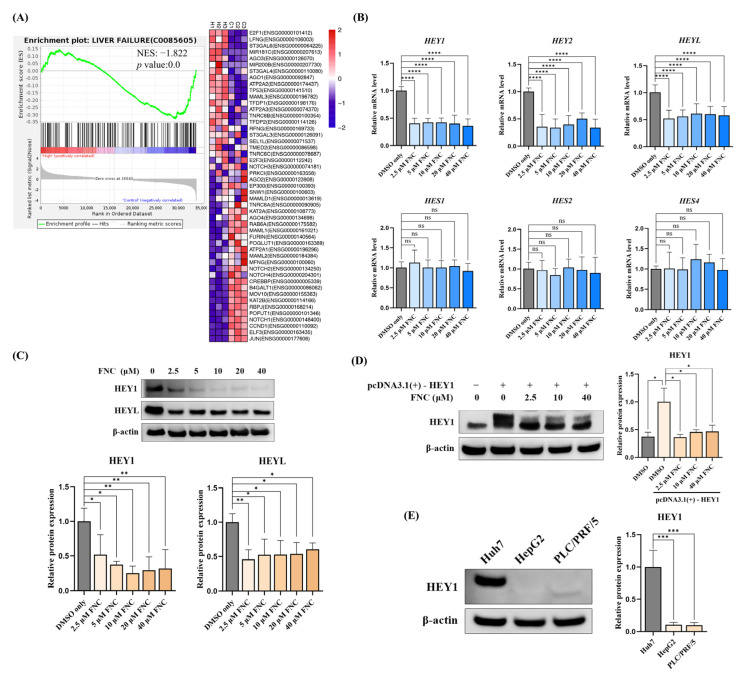
FNC inhibits the expression of HEY factors in Huh7 cell lines. (**A**) Enrichment plots showing changes in the Notch expression and processing gene set after FNC treatment in Huh7 cells. The heatmap displays relative gene expression levels normalized by row (gene); the colour gradient indicates the expression level and ranges from blue (lowest expression) to red (highest expression). (**B**) Total RNA was collected from Huh7 cells and analysed using real-time PCR after 48 h of FNC treatment. The control group was treated with DMSO only. A comparative Ct (ΔΔCt) analysis was performed to calculate the fold changes in mRNA expression relative to GAPDH. The experiment was independently performed in triplicate. (**C**) Protein expression levels of HEY1 and HEYL were analysed by Western blot after 48 h of FNC treatment at the indicated concentrations. β-actin was used as the loading control. Three independent experiments were performed, and a representative blot is shown. * *p* ≤ 0.05; ** *p* ≤ 0.01; *** *p* ≤ 0.001; **** *p* ≤ 0.0001. (**D**) HEY1 with a C-terminal Myc tag was overexpressed in Huh7 cells, followed by treatment with 2.5, 10 or 40 μM FNC. Three independent experiments were performed, and the data are presented as the mean ± SD. (**E**) HEY1 expression levels in Huh7, HepG2 and PLC/PRF/5 cells were detected by Western blot. Three independent experiments were performed. Data are presented as the mean ± SD. ns, no significant difference.

The RNA-Seq data suggest that the expression and processing of the Notch signalling pathway are significantly altered in FNC-treated Huh7 cells (Figure 5A). To investigate this further, we first analysed downstream regulators of the Notch signalling pathway. Strikingly, the real-time PCR results revealed that the transcription of the HEY family members, including HEY1, HEY2 and HEYL, were dramatically decreased in FNC-treated Huh7 cells compared to the cells treated with DMSO alone (Figure 5B). The Western blot results showed that the protein levels of HEY1 and HEYL were dramatically decreased in all FNC-treated samples (Figure 5C). However, no significant changes were observed in the mRNA levels of HES family members after 48 h of FNC treatment, but higher HES1 protein levels were detected in all FNC-treated groups compared to the control group after the same duration of treatment (Figure 5B and Figure S2).

HEY1 has been identified as a critical regulator of HCC and EMT in both clinical and laboratory studies [12,13,35]. To confirm that HEY1 is a target gene of FNC in Huh7 cells, we transiently overexpressed HEY1 with a C-terminal Myc tag using the pcDNA3.1(+) vector. Following transfection for 4 h, the cells were treated with 2.5, 10 or 40 μM FNC. Consistent with our hypothesis, Myc-tagged HEY1 overexpression was significantly inhibited in all FNC-treated groups compared to the overexpression sample without FNC treatment (Figure 5D). These findings suggest that HEY1 is a target of FNC and may play a vital role in the EMT inhibition process in Huh7 cells.

To investigate the possible cause of the differing EMT responses of HCC cell lines to FNC, we analysed HEY1 expression levels in Huh7, HepG2 and PLC/PRF/5 cells. The immunoblot data revealed that HEY1 expression in Huh7 cells was significantly higher than in HepG2 and PLC/PRF/5 cells (Figure 5E).

**Figure 6:**
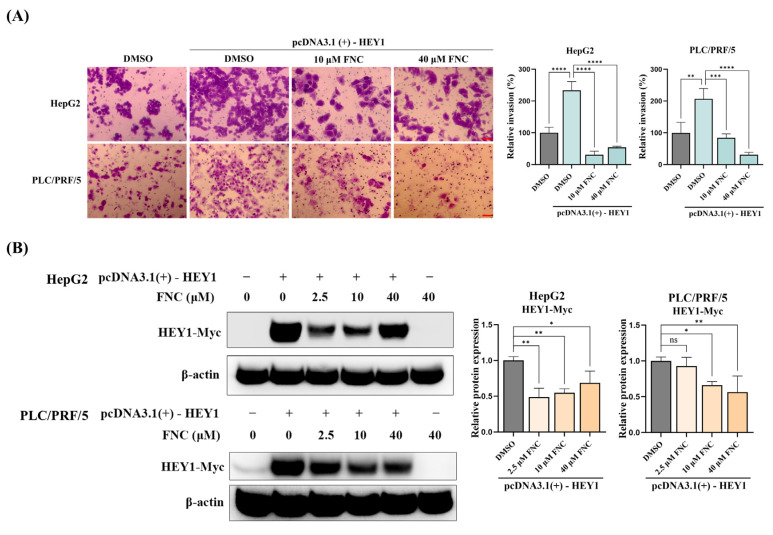
FNC inhibits HEY1-mediated invasion in HepG2 and PLC/PRF/5 cell lines. (**A**) HepG2 and PLC/PRF/5 cells transfected with C-terminal Myc-tagged HEY1 were treated with 10 or 40 μM FNC. Control cells were transfected with the empty pcDNA3.1(+) vector. Representative images show that HEY1 overexpression increased the invasive capacity of HepG2 and PLC/PRF/5 cells, and FNC treatment suppressed HEY1-driven invasion in both cell lines. Three independent experiments were performed, and data are presented as mean ± SD. Scale bar: 20 μm. (**B**) Western blot analysis showed that FNC suppresses Myc-tagged HEY1 protein levels in both cell lines. After 4 h of transfection, cells were treated with DMSO or FNC (2.5, 10, or 40 μM) for 48 h. Cell lysates were probed with an anti-Myc-HRP antibody, with β-actin used as the loading control. A representative Western blot from three independent biological replicates is shown. Data are presented as mean ± SD. * *p* ≤ 0.05; ** *p* ≤ 0.01; *** *p* ≤ 0.001; **** *p* ≤ 0.0001; ns, no significant difference.

To explore the heterogeneous responsiveness to FNC across the three HCC cell lines and its association with HEY1, we overexpressed C-terminal Myc-tagged HEY1 in HepG2 and PLC/PRF/5 cells prior to FNC treatment. Transwell invasion assays showed that HEY1 overexpression significantly enhanced the invasive capability of both HepG2 and PLC/PRF/5 cells (Figure 6A). Notably, both the 10 μM and 40 μM FNC treatments significantly suppressed HEY1-driven invasion compared to the DMSO-treated controls in both cell lines, with PLC/PRF/5 cells exhibiting a dose-dependent response (Figure 6A). The Western blot analysis demonstrated that FNC treatment significantly decreased HEY1 protein levels at all tested concentrations (2.5, 10 and 40 μM) in HepG2 cells, while higher doses (10 and 40 μM) were required to significantly reduce the levels in PLC/PRF/5 cells compared to the DMSO controls (Figure 6B). These findings indicate that HEY1 contributes to the heterogeneous responsiveness to FNC across HCC cell lines and represents a potential therapeutic target in HCC.

### 2.5. Molecular Docking, MST and MD Simulations Suggest That Jagged1 Is a Potential Direct Target of FNC

**Figure 7:**
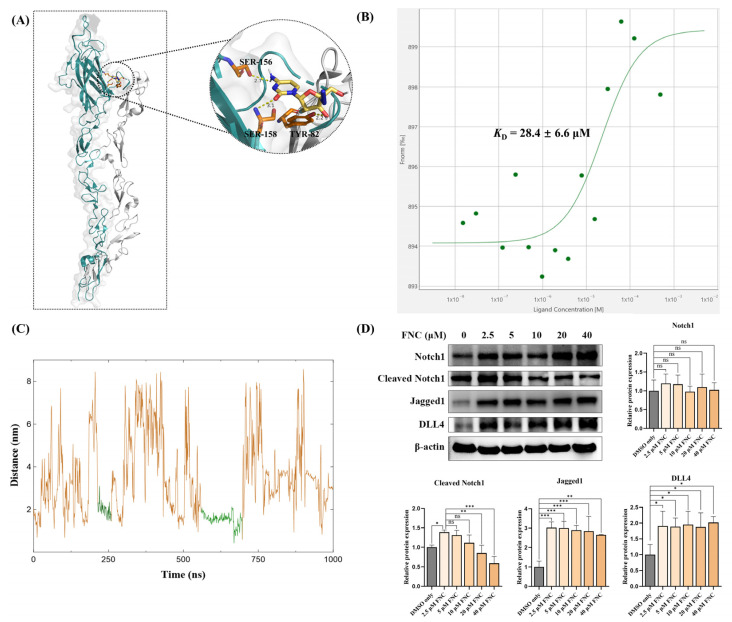
FNC interacts with Jagged1. (**A**) The interaction between FNC and Jagged1 (teal) as proposed by the molecular docking simulations. Notch1 (grey) was modelled onto the Jagged1 structure based on the Jagged1–Notch1 complex structure (PDB: 5UK5). Hydrogen-bond interactions with residues on the C2 domain are shown. (**B**) The binding of FNC to Jagged1-Fc was quantified in HEPES buffer. FNC was titrated at concentrations ranging from 0.015 to 500 μM, while the concentration of Jagged1-Fc was kept constant at 1 μM. A representative MST binding curve is shown. The K_D_ value is the average of triplicate measurements. (**C**) MD simulation of the Jagged1–FNC complex over a 1000 ns simulation. The MD trajectories show the protein-to-drug centre-of-mass distance. The green trajectories represent relatively stable conformations, indicating relatively consistent interactions between Jagged1 and FNC. (**D**) Jagged1, DLL4, Notch1 and cleaved Notch1 were detected by immunoblotting. Three independent experiments were performed, and the data were assessed using ordinary one-way ANOVA with comparisons to the mean of the 2.5 μM FNC-treated sample. ns, no significant difference; * *p* ≤ 0.05; ** *p* ≤ 0.01; *** *p* ≤ 0.001.

**Figure 8:**
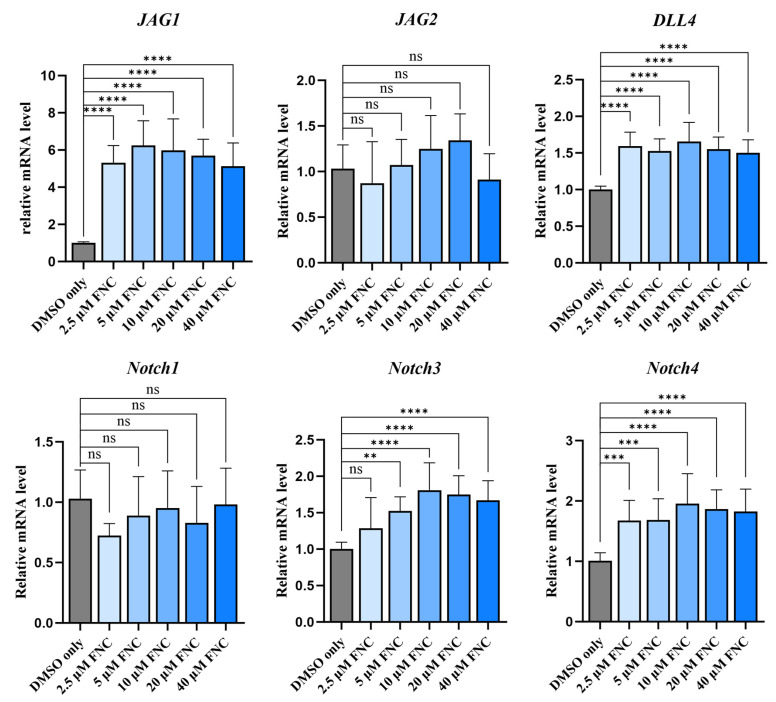
Effects of FNC on transcription levels of Notch ligands and receptors. Q-PCR analysis was performed to evaluate the transcription levels of Jagged1, Jagged2, DLL4, Notch1, Notch3 and Notch4 in Huh7 cells treated with 2.5, 5, 10, 20 and 40 μM FNC. Significant upregulation of Jagged1 and DLL4 mRNA levels were observed. Data represent the mean ± SD from three independent experiments. ** *p* ≤ 0.01; *** *p* ≤ 0.001; **** *p* ≤ 0.0001; ns, no significant difference.

To explore the mechanism underlying the HEY1 downregulation, we examined the transcriptional and expression levels of Notch ligands and receptors. The Western blotting and q-PCR data did not reveal significant decreases in the expression levels of any Notch ligand or receptor (Figure 7D and Figure 8). Interestingly, the transcriptional and expression levels of Jagged1 and DLL4 were increased in FNC-treated Huh7 cells compared to the control group (Figure 7D and Figure 8).

Since no significant decreases were observed in Notch ligand or receptor expression, we investigated whether FNC directly binds to Notch ligands and influences the Notch activation process. To predict the binding potential of FNC to Notch ligands, we utilized the AutoDock Vina 1.2.5 software to dock FNC to Jagged1, Jagged2, DLL1 and DLL4 (Table 1). Given that the core binding regions in the Notch ligands range from the C2 domain to EGF3, molecular docking was performed using the structure of this region. The binding energy, binding modes and binding residues were calculated to evaluate the interactions. The docking results revealed Jagged family ligands exhibited higher affinity for FNC compared to DLL family ligands, with Jagged1 having the lowest binding energy (−6.592 kcal/mol) (Table 1). The potential binding position of FNC on Jagged1 is shown in Figure 7A.

Building on the molecular docking results and prior studies indicating a strong correlation between Jagged1 and HEY1 expression, we next tested FNC’s binding affinity for Jagged1 using microscale thermophoresis (MST) [12,36,37]. Label-free MST assays were performed with recombinant human Jagged1 by utilizing its natural autofluorescence. The interaction of FNC with Jagged1 resulted in a dissociation constant (K_D_) of 28.4 ± 6.6 μM (Figure 7B).

To validate the molecular docking and MST results, molecular dynamics (MD) simulations of FNC binding to Jagged1 (C2 to EGF3, PDB:4CC1) were performed. A 1000 ns MD simulation was conducted, generating a trajectory of FNC’s distance from Jagged1 (Figure 7C). Various binding modes were identified, and the stability of these complexes was evaluated by monitoring the distance fluctuations throughout the simulation. The MD simulation results indicated that FNC binding to Jagged1 is a dynamic process. Two relatively stable binding conformations were identified: conformations 1 (around 250 ns) and 2 (around 600 ns). Conformation 1 suggests that FNC could bind to the DSL domain of Jagged1. Conformation 2 indicates that FNC preferentially binds to Jagged1 in its polymeric form, interacting with loops 1-2 of the C2 domain and EGF3 simultaneously. Furthermore, cleaved Notch1 levels were found to decrease in a dose-dependent manner following FNC treatment, which indicates that the activation of Notch1 was decreased in a dose-dependent manner following FNC treatment (Figure 7D). Interestingly, cleaved Notch1 levels increased in the 2.5 μM FNC-treated group compared to the DMSO-only group, potentially due to the increased Jagged1 expression after FNC treatment (Figure 7D and Figure 8). These findings, which are consistent with the MST results, suggest that FNC binding to Jagged1 may contribute to the downregulation of cleaved Notch1 and HEY1, which provides a potential mechanism for FNC-mediated inhibition of the Notch signalling pathway.

## 3. Discussion

FNC is a novel nucleotide analogue with antiviral and antitumour activities, which has been approved for the treatment of AIDS and COVID-19 [17]. In previous studies, FNC has been associated with the invasion of lymphoma cell lines [24] and the proliferation of various cancer cell lines [23].

Extensive evidence has shown that EMT plays a vital role in tumour metastasis, particularly in relation to tumour cell migration and invasion [9]. Previous studies have highlighted the plasticity between epithelial and mesenchymal states, where cancer cells were observed to be in the intermediate states along this spectrum [38]. In this study, we examined various types of cancer cell lines and demonstrated, for the first time, that FNC inhibits Huh7 cell migration and invasion in a dose-dependent manner (Figure 1A,B). Based on preclinical pharmacokinetic data showing that FNC plasma concentrations can reach ~2 µM in rhesus macaques [21], we treated Huh7 cells with FNC concentrations ranging from 2.5 µM to 40 µM to cover both the therapeutic and mechanistic investigation ranges. However, no anti-invasive effect was observed in HepG2 and PLC/PRF/5 cells with the 10 µM FNC treatment (Figure 1C), which likely reflects cell line-specific drug responses, intrinsic differences and degrees of invasiveness among HCC cell lines [39,40]. We further investigated the effects of FNC on EMT-related biomarkers in Huh7 cells and demonstrated that the transcriptional and translational levels of MMP2 and N-cadherin were decreased, accompanied by an upregulation of E-cadherin at the transcriptional level, but no change in the Vimentin and Snail levels (Figure 3A,B). The observed upregulation of E-cadherin mRNA without corresponding protein level changes suggests potential post-transcriptional regulation mechanisms, such as miRNA networks or protein stability modulations, were involved in this process [41]. Decreased transcriptional levels of MMP1 and MMP9 were also observed in Huh7 cells after FNC treatment. The changes in the levels of these MMPs may contribute to extracellular matrix remodelling, which could lead to the observed changes in cell shape (Figure 2E,F). These findings suggest a partial inhibition of EMT in Huh7 cells upon FNC treatment. Thus, FNC shows potential as a therapeutic agent for HCC by inhibiting the EMT process.

In the RNA-Seq experiments, we observed that FNC inhibits liver disease, liver failure, liver cirrhosis and fatty liver (alcoholic) gene sets according to the GSEA results (Figure 4B). Additionally, RNA-Seq identified differential expression of genes related to the Notch signalling pathway. Given that both HEY and HES family members are core transcriptional effectors of Notch signalling, we first validated these findings by analysing downstream regulators through q-PCR and Western blot. Our results suggested that FNC significantly inhibits multiple HEY factors, including HEY1, HEY2 and HEYL, indicating that they have a potential role in the EMT inhibition process. (Figure 5B,C). The inhibitory effect of FNC was further validated through HEY1 overexpression experiments (Figure 5D and Figure 6).

Previous studies have shown that HEY1 is an essential mediator of the TGF-β-induced EMT process in various cell types [12]. Clinical studies have also reported high expression of HEY factors in various cancers, highlighting their roles in tumour metastasis, angiogenesis as well as proliferation [37]. Furthermore, strong and growing evidence from clinical and basic research has underscored the importance of HEY1 in liver cancer development [13,35,37]. Therefore, HEY1 could be an interesting clinical target for liver cancer treatment. In our study, we tested the effects of FNC on three different HCC cell lines and observed that it exhibited anti-EMT activity in Huh7 cells. This may be due to the different expression profiles of the various HCC cell lines [42,43,44,45]. Given that HEY factors appear to be important targets of FNC in Huh7 cells, we further investigated whether the different responses to FNC were related to varying HEY factor expression levels. Our results, supported by data from the Human Protein Atlas database, indicated that Huh7 cells have higher HEY1 expression levels compared to the other tested cell lines, which may explain the observed anti-EMT effect of FNC on Huh7 cells [46,47] (Figure 5E). To validate this hypothesis, we overexpressed HEY1 in HepG2 and PLC/PRF/5 cells. The transwell invasion assays showed that HEY1 overexpression increased the invasiveness of both cell lines, and this effect was inhibited by FNC treatment (Figure 6A). The Western blot analysis confirmed that FNC inhibited exogenous HEY1 protein levels in both cell lines (Figure 6B). Collectively, these data suggest that HEY1 levels may be associated with the heterogeneous FNC responsiveness across HCC cell lines.

No significant changes were observed in the expression of Notch ligands or receptors (Figure 7D and Figure 8). To explore the mechanism underlying the HEY factor downregulation, molecular docking experiments were conducted to assess whether FNC binds to Notch ligands and potentially affects ligand-dependent Notch activation. Notch ligands and receptors have large ectodomains, many of which have been widely studied due to their critical role for Notch activation [48,49,50,51,52]. In addition to direct ligand–receptor interactions, some regions regulate Notch activation indirectly, such as the C2 domain of Notch ligands, which modulates Notch signalling through its lipid-binding properties [53,54]. Previous studies suggested that conformational changes in ligands and receptors during binding are crucial for Notch activation [51,52]. Our MST and MD simulations indicated that FNC directly interacts with Jagged1 (Figure 7B,C). The molecular docking results suggested that FNC might bind to the C2 domain of Jagged1, a critical domain for Notch activation due to its dual roles in Notch binding and lipid binding (Figure 7A). The binding of FNC to Jagged1 may induce conformational changes that alter the binding affinity of Jagged1 to Notch receptors and lipids, subsequently reducing Notch receptor activation and HEY1 expression. This hypothesis was further supported by detecting cleaved Notch1 which reflects the level of Notch1 activation. An increase in cleaved Notch1 was observed in Huh7 cells treated with 2.5 μM FNC compared to the DMSO-only group, likely due to the increased Notch ligand expression in the FNC-treated samples (Figure 7D and Figure 8).

Although small molecule–protein interaction identification approaches offer novel ways to identify potential drug targets, they also have many limitations [55]. MST is an in vitro assay that uses purified recombinant protein constructs. Although binding measurements were performed in a solution mimicking natural conditions, it cannot determine the binding behaviour of FNC to Jagged1 on liver cell surfaces or within the human body. Additionally, while the molecular docking and MD simulations identified potential binding sites for FNC on Jagged1, the complexity of the cell surface and tumour microenvironment coupled with the use of only part of Jagged1’s extracellular domain in the analyses limited our ability to determine the exact in vivo binding site.

Overall, our study provides novel insights into the potential of FNC for drug repurposing to treat hepatocellular carcinoma. We elucidated the mechanisms underlying FNC’s anti-EMT activity in HCC cell lines. Given its previously identified antiviral activities, including against HBV and HCV, FNC could be a valuable drug for HCC patients with HBV and HCV infections. Additionally, by investigating the heterogeneity between three HCC cell lines, we found that HEY1 was specifically elevated in Huh7 cells, suggesting its potential as a predictive biomarker for FNC responsiveness. Considering the high expression of HEY1 in a significant proportion of HCC patients, FNC may be a useful drug for these patients. While our findings suggest that FNC binding to Jagged1 may explain the decrease in HEY1 expression, further studies are needed to determine its effects on Jagged1-mediated Notch signalling and to identify the Jagged1-independent mechanisms responsible for HEY1 downregulation at lower FNC concentrations. Additionally, our study is limited by the lack of in vivo validation. Future work should investigate FNC in liver cancer animal models. In conclusion, our data indicate that FNC reduces the invasive and migratory capabilities of hepatocellular carcinoma cells by regulating Notch signalling and could have potential for therapeutic applications to treat hepatocellular carcinoma as well as Notch-associated disorders.

## 4. Materials and Methods

### 4.1. Cell Lines and Reagents

The hepatocellular carcinoma cell line Huh7 was obtained from the American Type Culture Collection (ATCC, Manassas, VA, USA). Huh7 cells were cultured in Roswell Park Memorial Institute (RPMI) 1640 medium supplemented with 10% foetal bovine serum (FBS), 100 U/mL penicillin and 100 μg/mL streptomycin. HepG2 and PLC/PRF/5 cells were cultured in Dulbecco’s Modified Eagle Medium (DMEM; Gibco, NY, USA) with 10% FBS, 100 U/mL penicillin and 100 μg/mL streptomycin. The cells were starved in a medium containing 0.1% FBS for 24 h prior to FNC treatment.

### 4.2. Cytotoxicity Assay

A 100 µL volume of a Huh7 cell suspension was inoculated at a density of 5 × 10^4^ cells/mL in a 96-well plate overnight. The culture medium was replaced with media containing different concentrations of FNC. After 48 h, 10 µL of a CCK-8 solution (MedChemExpress, NJ, USA) was added to each well. The plate was incubated for 1–4 h, and then the absorbance at a wavelength of 450 nm was measured using a plate reader (PerkinElmer 2030 multilabel Reader, VICTOR^TM^ X4; PerkinElmer, MA, USA).

The calculation formula was as follows:

Cell survival rate = [(As − Ab)/(Ac − Ab)] × 100%,(1)

where As = absorbance of the experimental well; Ac = absorbance of the control well; and Ab = absorbance of the blank well.

### 4.3. Wound-Healing Assay

Confluent, serum-deprived monolayer cultures of Huh7 cells were seeded into 6-well plates at a density of 1.2 × 10^5^ cells/mL. The cells were subjected to “wounding” by scratching the monolayer with a 10 µL plastic pipette tip. DMSO or varying concentrations of FNC were added to each well. After 24 and 48 h, cell migration was evaluated by measuring the open wound areas. Images were captured using a ZEISS Axio observer A1 inverted phase contrast microscope and analysed with Fiji software (Image J v2.14.0/1.54f).

### 4.4. Invasion Assay

Invasive activity was measured using BD Matrigel (BD Biosciences, Franklin Lakes, NJ, USA) in a 24-well plate following the manufacturer’s protocol. A total of 200 µL Huh7 cells in 0.1% FBS medium were seeded at a density of 0.8 × 10^4^ cells/mL into the upper chamber. The bottom wells of the system were filled with 400 µL of 10% FBS culture medium. After incubation for 24 or 48 h, the cells in the upper chamber were removed, and the cells on the bottom membrane were fixed and stained. The invading cells were counted in five random fields of the membrane. The invasion efficiency of the control cells was set as 100%.

### 4.5. RNA Sequencing

Huh7 cells were treated with 2.5 µM (low), 20 µM (medium) and 40 µM (high) FNC or DMSO only (control) for 48 h, with three biological replicates prepared for each sample group. The cells were lysed using TRIzol reagent (Invitrogen, Waltham, MA, USA) and sent to Novogene Services (Novegene Ltd, Cambridge, UK) for RNA library construction and sequencing. RNA integrity was assessed using the RNA Nano 6000 Assay Kit of the Bioanalyzer 2100 system (Agilent Technologies, Santa Clara, CA, USA). All samples demonstrated a high RNA integrity number (RIN) index > 5, qualifying them for further analysis. The library preparations were sequenced on an Illumina NovaSeq platform, generating 150 bp paired-end reads.

Differential expression analysis was performed using the DESeq2 R package (v1.20.0). Genes with an adjusted *p*-value < 0.05 were classified as differentially expressed. Gene Set Enrichment Analysis (GSEA) was conducted using DisGeNET datasets. Genes were ranked by the degree of differential expression between the two samples, and predefined gene sets were tested for enrichment at the top or bottom of the ranked list. Enrichment results with *p* < 0.05 were considered statistically significant.

### 4.6. Western Blotting

The cells were washed with PBS before lysis in ice-cold lysis buffer (P0013C, Beyotime, Haimen, China). The protein concentrations were measured using the Pierce^TM^ BCA Protein Assay Kit (Thermo Fisher Scientific, Waltham, MA, USA) after sample lysis. The lysates were then subjected to SDS-PAGE, followed by immunoblotting. The following antibodies were used: anti-β-actin (13E5, Cell Signaling Technology, Danvers, MA, USA), anti-MMP2 (D4M2N, Cell Signaling Technology), anti-MMP9 (D6O3H, Cell Signaling Technology), anti-N-cadherin (D4R1H, Cell Signaling Technology), anti-E-cadherin (24E10, Cell Signaling Technology), anti-HEY1 (ab154077, Abcam, Cambridge, UK), anti-HES1 (D6P2U, Cell Signaling Technology), anti-Vimentin (D21H3, Cell Signaling Technology) and anti-Snail (C15D3, Cell Signaling Technology). Detection was performed using a chemiluminescence detection system (Bio-Rad ChemiDoc^TM^, Hercules, CA, USA).

### 4.7. Real-Time PCR

Total RNA from the Huh7 cell line was isolated using an RNA isolation kit (TransGen Biotech, Beijing, China). Reverse transcription was performed with the High-Capacity cDNA Reverse Transcription Kit (Applied Biosystems^TM^, Waltham, MA, USA). The reactions were run using a standard SYBR Green Master Mix (Vazyme, Nanjing, China) in 96-well plates (Applied Biosystems^TM^) on an Analytik Jena qTOWER^3^ RT-PCR system. The comparative Ct (ΔΔCt) method was applied to determine the fold change in mRNA expression with glyceraldehyde-3-phosphate dehydrogenase (GAPDH) used as the reference gene. Each sample was run in triplicate. All primers used in this study are listed in Supplementary Table S1.

### 4.8. Immunofluorescence Microscopy

Cells were grown in 6-well plates containing coverslips for 24 h before being treated with 40 µM FNC or DMSO for 48 h. After treatment, the cells were fixed with 4% paraformaldehyde for 30 min at room temperature and permeabilized with 0.1% TritonX-100 in PBS for 10 min on ice. The cells were then rinsed three times with PBS and blocked with 5% BSA for 1 h at room temperature. The cells were incubated with the indicated antibodies diluted in PBS overnight at 4 °C. After washing with PBS, the cells were incubated with Alexa Fluor 488-conjugated anti-rabbit antibodies for 1 h at room temperature. Finally, the coverslips were placed onto slides using SlowFade^TM^ Diamond Antifade Mountant with DAPI (Invitrogen^TM^). The samples were imaged using a Nikon Ti2U fluorescence microscope. The following antibody was used: Phalloidin iFluor^TM^ 488 (YEASEN, Shanghai, China).

### 4.9. MST

MST traces were generated using a Monolith NT.LabelFree instrument (Nano Temper Technologies, München, Germany) with the MO.Control v2.0.4 software. A full-length extracellular domain (C2 to EGF16, 1277-JG-050, R&D Systems) tagged with an Fc-tag (Jagged1-Fc) was expressed in Chinese hamster ovary (CHO) cells. Jagged1-Fc was dissolved in HEPES buffer (20 mM Hepes (pH 7.4), 150 mM NaCl and 2 mM CaCl_2_) to a concentration of 1 μM. FNC was serially diluted in HEPES buffer to concentrations ranging from 0.015 to 500 μM. Jagged1-Fc was then mixed 1:1 (v/v) with the FNC solutions. The mixtures were incubated for 15 min at room temperature. The samples were then transferred into LabelFree Premium Capillaries (NanoTemper Technologies) and analysed at 12% excitation power on the Monolith NT.LabelFree instrument at a constant temperature of 25 °C. The dissociation constant (*K*_D_) was determined as the average of three biological replicate values, which were obtained using the MO.Control software v1.6.1.

### 4.10. Molecular Docking

Protein receptor structures were sourced from the RCSB Protein Data Bank (PDB). Protein macromolecules and the 3D structure of FNC were imported into AutoDock Tools 1.5.6. Water molecules were removed, hydrogen atoms were added and the structures were converted into PDBQT format. The grid box was configured to encompass the entire receptor region. Semi-flexible docking was performed using AutoDock Vina to evaluate the interaction forces between FNC and the protein receptors. The 3D conformation of the ligand–receptor complex was visualized using PyMOL 3.0.

### 4.11. MD Simulations

The three-dimensional structure of Jagged1 (PDB: 4CC1) was obtained from the Protein Data Bank. MD simulations were performed using GROMACS 2020.6 software [56,57,58] and applying the AMBER99SB-ILDN force field [59]. The small molecule was prepared using Sobtop [60] to generate a GAFF force field, with force parameters for the azide structure calculated using the Hessian matrix. A cubic simulation cell, 0.8 nm larger than the FNC–Jagged1 complexes in all dimensions, was employed. Water molecules were modelled using the TIP3P model at a density of 1 kg/L, and the system was neutralized with Na^+^ and Cl^−^ ions. The system’s temperature was gradually increased to 298.15 K over 500 ps. Free dynamic simulations were then conducted using the Verlet algorithm with a 0.002 ps integration time step. The simulations were performed in an isothermal–isobaric (NPT) ensemble at 298.15 K and 1 bar pressure, with the temperature and pressure controlled using the V-rescale and Parrinello–Rahman methods, respectively. Periodic boundary conditions were applied throughout the simulations, and the duration was extended as needed.

Root mean squared deviation (RMSD) values were calculated to assess the Jagged1–FNC interactions. MD trajectories were visualized using VMD software version 1.9.4. The binding free energy of the Jagged1–FNC complex was calculated using the gmx_MMPBSA package and the molecular mechanics/Poisson–Boltzmann surface area (MM/PBSA) method.

### 4.12. Statistical Analysis

Statistical analyses were performed using Prism 10 (GraphPad, San Diego, CA, USA). The experiments were repeated at least three times, and the data are presented as the mean ± standard deviation (SD) unless otherwise stated. *p*-values were calculated using ordinary one-way ANOVA, followed by Dunnett’s post hoc test to analyse the differences between each pair of groups. A *p*-value of less than 0.05 was considered statistically significant. For the RNA-Seq analysis, the resulting *p*-values were adjusted using the Benjamini–Hochberg method to control the false discovery rate.

## Figures and Tables

**Table 1:** Binding interactions and energies between Notch ligands and FNC as proposed by the molecular docking simulations.

Protein *	Residue	Binding Mode	Binding Energy (kcal/mol)
Jagged1	TYR 82	H-Bond	−6.592
	SER 156	H-Bond	
	SER 158	H-Bond	
	VAL 86	Pi-A	
Jagged2	SER 204	SER 204	−6.506
	ARG 114	ARG 114	
	CYS 198	CYS 198	
	ASP 176	ASP 176	
	TYR 203	TYR 203	
DLL1	HIS 182	H-Bond	−5.707
	ASP 180	H-Bond	
	TYR 183	H-Bond	
	GLY 185	H-Bond	
	CYS 179	Hal/Pi-S	
DLL4	GLN 153	H-Bond	−6.058
	CYS 175	H-Bond	
	GLY 181	H-Bond	
	ASP 182	H-Bond/Attr-Chg	
	PRO 202	C-H Bond	

* Jagged1 PDB = 4CC1; Jagged2 PDB = 5MW7; DLL1 PDB = 4XBM; DLL4 PDB = 5MVX.

## Data Availability

The data presented in this study are available on request from the corresponding author.

## Supplementary Materials

The following supporting information can be downloaded at: https://www.mdpi.com/article/10.3390/ijms26115127/s1.

## Abbreviations

The following abbreviations are used in this manuscript:

H-Bond	Hydrogen Bond
Pi-A	Pi-Alkyl
Pi-Cat	Pi-Cation
Hal	Halogen
C-H Bond	Carbon Hydrogen Bond
Pi-Pi	Pi-Pi Stacked
Pi-S	Pi-Sulphur
Attr-Chg	Attractive Charge
HIS	Histidine
TYR	Tyrosine
SER	Serine
VAL	Valine
ARG	Arginine
CYS	Cysteine
ASP	Aspartic Acid
GLY	Glycine
GLN	Glutamine
PRO	Proline
