# Supplementary material for: Azvudine Suppresses Epithelial–Mesenchymal Transition in Hepatocellular Carcinoma by Targeting the Notch–HEY Signalling Pathway
Source: Int J Mol Sci. 2025 May 27;26(11):5127. doi: 10.3390/ijms26115127 (PMC12154575; doi:10.3390/ijms26115127)
Supplement: Supplementary file 1 [file ijms-26-05127-s001.zip › ijms-3567582-supplementary.pdf]

International Journal of Molecular Sciences

MDPI

Supplementary

Table S1. List of primers used in this paper.

Gene	Primer	Sequence (5′-3′)
CDH1	Forward	CGAGAGCTACACGTTCACGG
	Reverse	GGGTGTCGAGGGAAAAATAGG
CDH2	Forward	TTTGATGGAGGTCTCCTAACACC
	Reverse	ACGTTTAACACGTTGGAAATGTG
VIM	Forward	TGACCGCTTCGCCAACTA
	Reverse	CCCGCATCTCCTCCTCGTA
SNAIL	Forward	TCGGAAGCCTAACTACAGCGA
	Reverse	AGATGAGCATTGGCAGCGAG
MMP1	Forward	AAAATTACACGCCAGATTTGCC
	Reverse	GGTGTGACATTACTCCAGAGTTG
MMP2	Forward	TACAGGATCATTGGCTACACACC
	Reverse	GGTCACATCGCTCCAGACT
MMP9	Forward	TGTACCGCTATGGTTACACTCG
	Reverse	GGCAGGGACAGTTGCTTCT
HEY1	Forward	CATACGGCAGGAGGGAAAG
	Reverse	GCATCTAGTCCTTCAATGATGCT
HEY2	Forward	CCCGCCCTTGTCAGTATC
	Reverse	TTGTTTGTTCCACTGCTGGT
HEYL	Forward	GTCCCCACTGCCTTTGAG
	Reverse	ACCGTCATCTGCAAGACCTC
HES1	Forward	GAAGCACCTCCGGAACCT
	Reverse	GTCACCTCGTTCATGCACTC
HES2	Forward	CAGCTTAAGGGGCTCATCCT
	Reverse	GGACGTCTGCCTTCTCTAGC
HES4	Forward	CTGGACGCCCTCAGAAAA
	Reverse	GCTCCGCAGGTGTCTCAC
Jagged1	Forward	GAATGGCAACAAAACTTGCAT
	Reverse	AGCCTTGTCGGCAAATAGC
Jagged2	Forward	GCCTGGCCGCGTTCTTT′
	Reverse	AGCTCCTCATCTGGAGTGGT
DLL4	Forward	CCCTGGCAATGTACTTGTGAT
	Reverse	TGGTGGGTGCAGTAGTTGAG
Notch1	Forward	CACCAGGGTGGTCAGGAAAA
	Reverse	GGGCAGCGACAGATGTATGA
Notch3	Forward	ACTCCTCCTCAGGGAGATGC
	Reverse	GTGGGGTGAAGCCATCAGG
Notch4	Forward	ACAAGGGATCTGCCAGTGTG
	Reverse	CAGGTGCAGGAGAAGTGAGG
GAPDH	Forward	AGCCACATCGCTCAGACAC
	Reverse	GCCCAATACGACCAAATCC

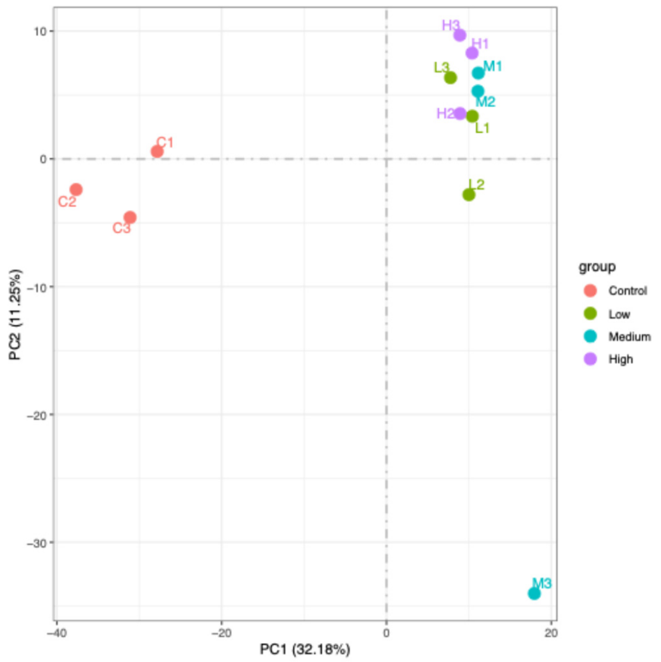

Figure S1. Principle component analysis (PCA) plot of samples treated with low, medium and high concentrations of FNC and the control group. The control group is shown in red, while the low-, medium-, and high-concentration FNC-treated groups are shown in green, blue and purple, respectively. The PCA plot demonstrates the separation of the samples based on the FNC treatment concentration, with PC1 and PC2 accounting for 32.18% and 11.25% of the total variance, respectively.

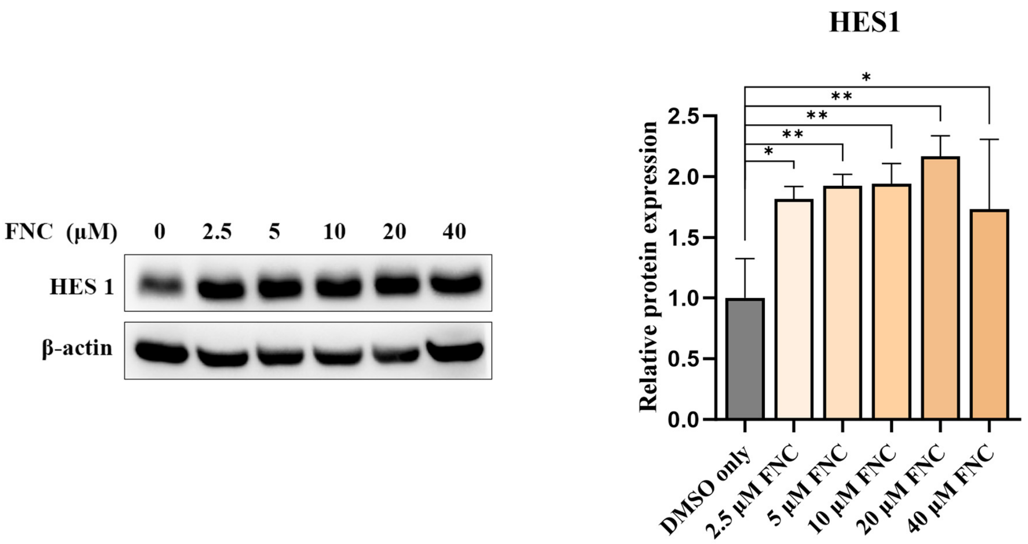

Figure S2. FNC upregulates HES1 protein expression in Huh7 cells. Western blot analysis showing the expression of HES1 protein in Huh7 cells treated with 2.5, 5, 10, 20 and 40 μM FNC. The control group (DMSO treatment only) and FNC-treated groups were compared. The FNC treatment resulted in an upregulation of HES1 protein expression. Data were obtained from three independent experiments, and a representative figure is shown.
